# Identification of circular RNAs in the ovarian follicles of Meishan and Duroc sows during the follicular phase

**DOI:** 10.1186/s13048-020-00709-5

**Published:** 2020-09-11

**Authors:** Su Xie, Mengxun Li, Yansen Chen, Yi Liu, Lipeng Ma, Xiaomei Sun, Yishan Sun, Ruonan Gao, Tao Huang

**Affiliations:** 1 College of Animal Science and Technology, Shihezi University, 221 North Fourth Road, Shihezi, 832000 China; 2 Key Laboratory of Animal Breeding and Reproduction of Minstry of Education,College of Animal Science and Technology, Huazhong Agricultural University, Wuhan, 430070 China; 3 University of Liège, Gembloux Agro-Bio Tech (ULiège-GxABT), Gembloux, Belgium

**Keywords:** Middle-sized ovarian follicles, Sows, Circular RNAs, Follicular phase, Meishan

## Abstract

Circular RNAs (circRNAs) are a newly discovered class of endogenous non-coding RNAs that play an important role in growth and development by regulating gene expression and participating in a variety of biological processes. However, the role of circRNAs in porcine follicles remains unclear. Therefore, this study examined middle-sized ovarian follicles obtained from Meishan and Duroc sows at day 4 of the follicular phase. High-throughput RNA sequencing (RNA-seq) was utilized to construct circRNAs, and differential expression was identified. The findings were validated using reverse transcription PCR (RT-PCR) and DNA sequencing, GO and KEGG analyses were performed, and potential miRNA targets were identified. The RNA-seq identified a total of 15,866 circRNAs, with 244 differentially expressed in the Meishan relative to the Duroc (111 up-regulated and 133 down-regulated). The RT-PCR finding confirmed the RNA-seq results, and quantitative real-time PCR (qPCR) analysis examining a subset of the circRNAs showed that they are resistant to RNase R digestion. Bioinformatics analysis (GO and KEGG) showed that the host genes associated with the differentially expressed circRNAs are involved in reproduction and follicular development signaling pathways. Furthermore, many of the circRNAs were found to interact with miRNAs that are associated with follicular development. This study presents a new perspective for studying circRNAs and provides a valuable resource for further examination into the potential roles of circRNAs in porcine follicular development.

## Introduction

For the past few years, circular RNAs (circRNAs) have attracted attention as a new member of the noncoding RNA family in animals [1–3]. In 1976, Sanger et al. first discovered that certain higher plant viruses were covalently closed circular RNA molecules [4]. Then in 1986, Kos et al. discovered that the hepatitis delta virus (HDV) genome comprises circRNA [5]. In recent years, breakthroughs in high-throughput deep sequencing technology have identified circRNAs in humans [3, 6, 7], mice [6, 7], nematodes [7, 8] and coelacanths [9]. Furthermore, studies have shown that circRNAs exhibit specific expression patterns in different tissues or cell types and at different developmental stages [3, 7]. The richness and diversity of circRNA expression may be related to the alternative splicing of RNA transcripts. Moreover, circRNA formation is facilitated by the complementary pairing between repeated intron sequences on both sides of axons [10–13]. Recent circRNA studies have shown that circRNAs possess structural diversity, a high abundance, and a high resistance to exonuclease or RNase degradation, are highly conserved, and have cell or tissue specific expression [13–15].

However, few studies have examined the regulation of circRNAs in association with animal reproduction. When examining ovary, testis, and placental circRNA expression patterns, studies have suggested a role in regulating the reproductive system and embryo development [16–19]. In a study performing transcriptome analysis of mouse germline cells, 18,822 circRNAs were identified, with 921 being sex related [20]. In another study examining differential circRNA expression in placenta tissues in pregnant women with preeclampsia (PE), a total of 143 up-regulated and 158 down-regulated circRNAs were identified [17]. Additionally, recent evidence suggests that circRNAs are involved in a wide range of biological processes and function as competitive endogenous RNAs (ceRNA) [21, 22]. During the cell cycle, circular RNA FoxO3 interacts with CKD2 (cyclin-dependent protein kinase 2) to stop the cell cycle progression during the G1/S phase [23]. CircRNAs have also been shown to regulate gene expression at the post-transcriptional level by binding to micro-RNAs (miRNAs), with cdr1as shown to act as a potent miRNA sponge that binds mir-7 and facilitates mRNA preservation [23, 24].

In the swine industry, sow productivity is one of the most important factors affecting the production efficiency. The main factor that limits litter size is the ovulation rate, with a higher ovulation rate contributing to a larger litter size. In China, the Meishan breed, a sub-group of the Taihu pig, provides sows with larger litter sizes relative to Durocs and is known for its high fecundity. In a previous study, differences in follicular growth dynamics between prolific Meishan sows and ordinary sows were found to occur during the mid-to-late-follicular phase and greatly contributed to the high ovulation rate [25]. Furthermore, another study suggested that there are significant differences in middle-size ovarian follicle growth regulation and physiological development between Meishan and Duroc sows [26]. Additionally, circRNAs have been examined in other organisms, including Drosophila ovarian tissue [27], goat ovarian follicles prior to ovulation [28], and honeybee ovaries in association with activation and spawning [29]. However, circRNAs have not been examined in association with porcine follicular development. Therefore, this study utilized RNA sequencing (RNA-seq) to explore circRNA differential expression during follicular development in Meishan and Duroc pigs. It is hoped that the results of this study will provide insight into the potential function of circRNA in porcine follicular development and aid in identifying circRNAs that are key to this process.

## Materials and methods

### Ethics statement

All procedures involving animals were approved by the Animal Care Committee of Shihezi University (Shihezi, China) and were conducted in accordance with the ethical standards established in the 1964 Declaration of Helsinki and its subsequent amendments.

### Tissue sample collection

Meishan sows (*n* = 3) were obtained from Yangzhou University (Yangzhou, Jiangsu, China), and Duroc sows (n = 3) were obtained from Tiankang Animal Husbandry Co., Ltd. (Xinjiang, China). All multiparous sows showed a normal estrus and reproductive performance in accordance with their breed characteristics. A day 14 of estrus, veterinary chloroprostol (0.2 mg/per pig) was injected along the ear vein, and the sows were subsequently slaughtered 4 days later. The M2 follicles (~ 5.0–6.9 mm in diameter) were collected and snap frozen in liquid nitrogen. Total RNAs were isolated using TRIzol (Invitrogen, CA, USA) according to the manufacturer’s protocol, and RNA quantity and purity were assessed using a RNA6000 Nano Kit and Bioanalyzer 2100 (Agilent, CA, USA).

### RNA-seq and quality control

Prior to RNA-seq, ribosomal RNA was removed from each individual sample (3 μg) using an Epicentre Ribo-Ribo-zero™ rRNA Removal Kit (Epicentre, USA), followed by ethanol precipitation to clean-up the rRNA free residue. Sequencing libraries were then generated using the rRNA-depleted RNA and a NEBNext® Ultra™ Directional RNA Library Prep Kit for Illumina® (NEB, USA) according to the manufacturer’s recommendations. Briefly, fragmentation was carried out in NEBNext First Strand Synthesis Reaction Buffer (5X) using divalent cations under an elevated temperature. First strand cDNA synthesis was performed using random hexamer primers and M-MuLV Reverse Transcriptase (RNaseH), and second strand synthesis was performed using DNA Polymerase I and RNase H, with remaining overhangs converted to blunt ends. For the dNTPs in the reaction buffer, dTTPs were replaced with dUTPs. After performing polyadenylation, a NEBNext Adaptor with hairpin loop structure was ligated to prepare for hybridization. To select cDNA fragments of a preferred length (~ 150–200 bp), the library fragments were purified using an AMPure XP system (Beckman Coulter, Beverly, MA, USA). The obtained size-selected, adaptor-ligated cDNA was then combined with 3 μl USER Enzyme (NEB) at 37 °C for 15 min, followed by 5 min at 95 °C. The PCR reaction was then performed with Phusion High-Fidelity DNA polymerase (NEB), Universal PCR primers, and Index (X) Primer. Finally, the PCR products were purified (AMPure XP system) and the library quality was assessed using an Agilent Bioanalyzer 2100 system. Index-coded samples were clustered using a TruSeq PE Cluster Kit v3-cBot-HS (Illumia, San Diego, CA, USA) with a cBot Cluster Generation System according to the manufacturer’s instructions. The libraries were then sequenced on an Illumina Hiseq 2500 platform, and 125 bp paired-end reads were generated. Raw data (raw reads) were obtained in FASTQ format and were preliminarily processed through an in-house perl script. In this step, clean reads were obtained by removing reads containing adapters or ploy-N and by removing low quality reads. Next, Q20, Q30, and GC contents were calculated for the clean reads, and all downstream analyses were performed using this high-quality clean data.

### CircRNA identification

Reference genome and gene model annotation files were downloaded directly from the genome website (http:/genome.ucsc.edu/). An index for the reference genome was built using bowtie v2.2.8, and paired-end clean reads were aligned to the reference genome using TopHat v2.0.9. The circRNAs were detected and identified using find_circ [7]. The basic principle of find_circ is to extract a 20 nt anchor sequence from each end of a given read without comparison to the reference sequence, and then compare each pair of anchor sequences to the reference sequence. A read was determined to be a candidate circRNA if the 5′ end of the anchor sequence was aligned to the reference sequence (the start and stop sites are labeled A3 and A4, respectively), if the 3′ end of the anchor sequence was aligned upstream of that site (the start and stop sites are labeled A1 and A2, respectively), and if a splice site (GT-AG) existed between A2 and A3 in the reference sequence. Finally, a candidate circRNA was confirmed as a circRNA if the read count is greater than or equal to 2

### Analysis of differentially expressed circRNAs

The raw counts were first normalized using TPM (transcripts-per-million clean tags) [30], and differential expression analysis was performed using the R package DESeq (1.10.1). DESeq provides statistical routines for determining differential expression for digital gene expression data using a model based on the negative binomial distribution. CircRNAs with a *p* < 0.05 and |log2 (fold change)| > 1.5 were considered significantly differentially expressed.

### Target site prediction and enrichment analysis

MicroRNA target sites were identified within the exons of circRNA loci and were identified using miRanda and psRobot. Gene Ontology (GO) enrichment analysis was employed to characterize the host genes of the differentially expressed circRNAs using DAVID [31]. GO terms with a corrected *p*-value less than 0.05 were considered significantly enriched. Differential gene expression and circRNA host genes were further examined using KEGG pathway analysis, and statistical enrichment was determined using KOBAS [32]. Findings were considered statistically significant if *p* < 0.05 was obtained.

### Reverse transcription PCR (RT-PCR) analysis and sequencing

Total RNAs were extracted from the sow ovarian follicles using TRIzol (Invitrogen, CA, USA), and cDNA was synthesized using a RT-PCR kit (Takara, Dalian, China). The PCR reaction was conducted using specific primers for circ_0012855, circ_0001712, circ_0015292, circ_0001651, circ_0008449, circ_0012124, circ_0000058, circ_0003666, circ_0010513, and circ_0011249 (Table 1). The PCR products were analyzed by gel electrophoresis, and a direct gene sequence analysis was performed. The PCR product sequences were then compared with the *Sus scrofa* reference genome and the RNA-seq data using DNAMAN software. The PCR reaction was conducted by combining 10 μL of premix (Takara, Dalian, China), 1 μL of cDNA template, 0.6 μL each of upstream and downstream primers, and 7.8 μL of RNase-free ddH_2_O water. RT-PCR was performed as follows: an initial denaturation at 95 °C for 5 min, followed by 45 cycles at 95 °C for 30 s, Tm (°C) for 30 s, and 72 °C for 30 s.

**Table 1:** List of RT-PCR primers

CircRNA	Primer sequences (5′–3′)	PCR products (bp)
GAPDH	F: TTCCAGTATGATTCCACCCACG	242
	R: TCGGCAGAAGGGGCAGAGAT	
novel_circ_0012855	F: CCCAAAGTGGCAACAAGG	168
	R: CGGTTCACAGATGAGGAGG	
novel_circ_0001712	F: GGCTTCAGCATCATCCCT	156
	R: TCGCTCGGTCTCCCATTT	
novel_circ_0015292	F: CACTGTGCCTCCTTGGGG	169
	R: CCAACCAGAGTGTATCCTTCATC	
novel_circ_0001651	F: GACGAGATGAGCGATGTGG	146
	R: GACGGGTTCTGGATGTGC	
novel_circ_0012124	F: TCTTTGTGTATTTCTGCCTG	147
	R: CCTTGATTTTCCTTGTCCT	
novel_circ_0010513	F: TCACAAATAAAGCCATCAGC	182
	R: ATACCGAATGCCCGAAAG	
novel_circ_0011249	F: CTGAGCGGTGTGTGTTCG	164
	R: GGCATTGGTGTCGTTGGT	
novel_circ_0000058	F: AGCAGAGTCTGTGATGCAGG	136
	R: GCTCGATCCCGATCAAATGC	
novel_circ_0003666	F: GAAGAATGTTTTCAGGCCG	143
	R: CTGCTCATTTTCTACATCCA	
novel_circ_0008449	F: GCAATTACCGTCCCAGGAGGA	139
	R: CTGAAGATGGTGGGGGATTGA	

### Quantitative real-time PCR (qPCR) analysis

The expression levels of four circRNAs (circ_0015292, circ_0001651, circ_0012124, and circ_0010513) were detected via qPCR analysis, with GAPDH used as an internal reference gene [33]. To determine the resistance of circRNAs to RNase R digestion, total RNAs were treated with RNase R (RNR-07250; Epicentre) prior to cDNA synthesis. To validate differentially expressed circRNAs, total RNAs were directly subjected to cDNA synthesis using a RT-PCR kit (Takara, Dalian, China). Reactions were performed using SYBR Green I (TaKaRa Biotech, Dalian) according to the manufacturer’s protocol, and circRNA expression levels were normalized to linear GAPDH levels. Three independent experiments were performed using triplicate samples. The qPCR reaction was conducted by combining 10 μL of SYBR Premix DimerEraser (Takara, Dalian, China), 1 μL of cDNA, 0.6 μL each of the upstream and downstream primers, and 7.8 μL of RNase-free ddH_2_O water. The qPCR reaction was performed as follows: an initial denaturation at 95 °C for 300 s, followed by 45 cycles at 95 °C for 30 s, Tm (°C) for 30 s, and 72 °C for 30 s.

## Results

### High-throughput sequencing of porcine ovarian follicle circRNAs

**Fig. 1:**
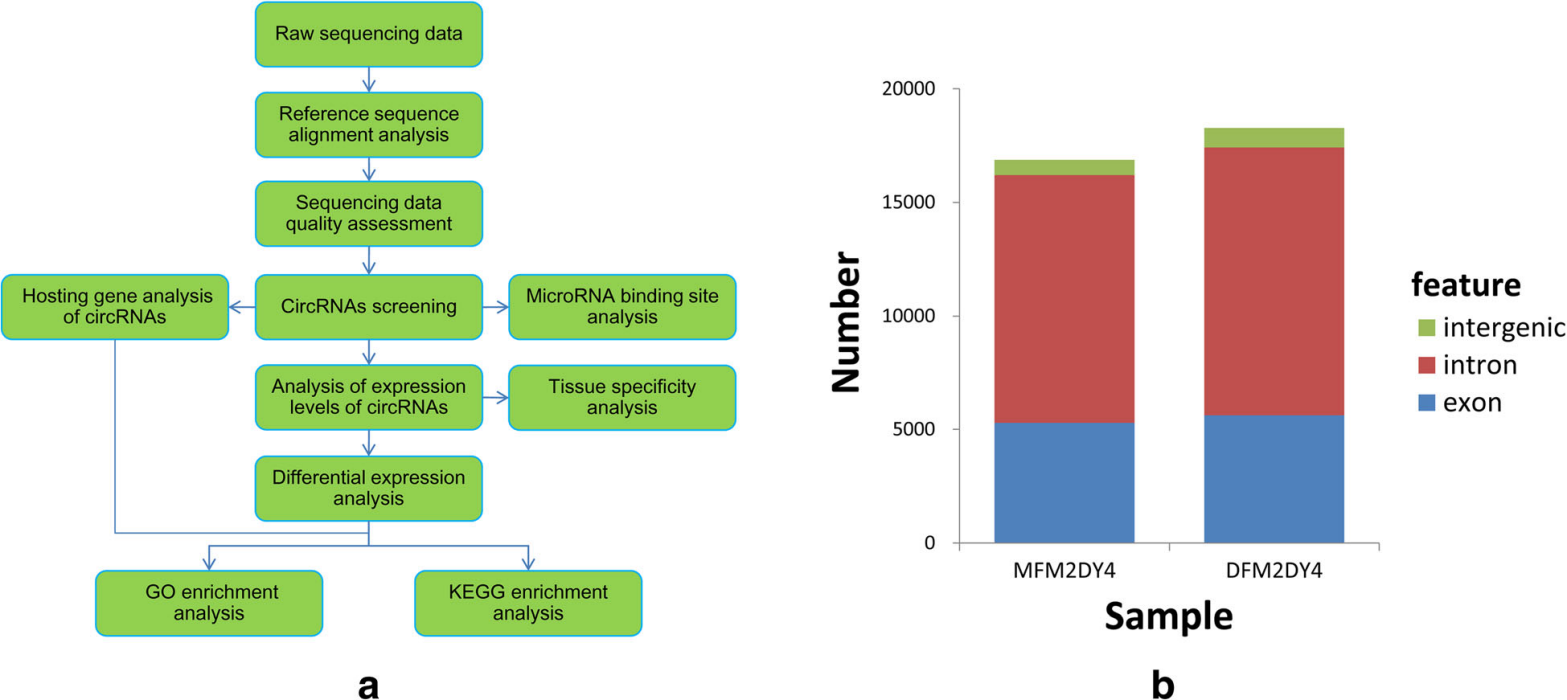
Swine follicle circRNA identification and structural analysis. **a** CircRNA identification procedure; **b** structural analysis of circRNAs

To determine circRNA identities and abundances in intermediate porcine follicles obtained from Meishan and Duroc pigs, RNA-seq was performed (Fig. 1*a*) and circRNAs within the two libraries were identified using the find_circ program [7]. A total of 15,866 circRNAs were identified between the two groups and were found to consist of introns, exons, and a small number of intergenic sequences (Fig. 1*b*). Additionally, circRNA annotations, chromosomal locations, and host mRNA were also determined, with the top 25 up- and down-regulated circRNAs also identified based on log2FC values (Table 2).

**Table 2:** Top 25 significantly up-regulated and down-regulated ovarian follicle circRNAs in Meishan sows relative to Duroc sows

Top 25 up-regulated				Top 25 down-regulated			
ID	log2FC	*p*	gene symbol	ID	log2FC	*p*	gene symbol
circ_0001651	7.1574	4.73E-06	TUBG1	circ_0015292	−7.6042	6.84E-07	C4BPA
circ_0010513	6.2571	0.000149	SLC44A3	circ_0012124	−7.0032	8.38E-06	YBX1
circ_0012307	5.8764	0.000529	CIC	circ_0001712	−6.854	1.58E-05	SNF8
circ_0000058	5.3764	0.002057	TRIP12	circ_0001710	−6.5953	4.22E-05	UBE2Z
circ_0008587	5.339	0.002296	TCP11L1	circ_0011249	−6.1634	0.00019289	ARFGAP3
circ_0009283	4.981	0.005482	PLEKHH2	circ_0001727	−5.8201	0.00057745	ACSF2
circ_0010364	4.7663	0.009321	PDE4DIP	circ_0015096	−5.8196	0.00057511	PPP2R1B
circ_0008763	4.7319	0.012184	SNAP47	circ_0000779	−5.6115	0.0010579	ERCC6L2
circ_0013004	4.6823	0.010512	FLVCR2	circ_0004779	−5.3668	0.0020519	COPS7B
circ_0014139	4.6314	0.015421	APBB2	circ_0011788	−5.2487	0.0027847	OSBPL1A
circ_0015559	4.6267	0.014702	CT55	circ_0014246	−5.1531	0.0035303	TMEM128
circ_0006839	4.6253	0.011693	FAN1	circ_0010747	−4.9044	0.0068792	RGS22
circ_0003799	4.582	0.012761	FAM53B	circ_0012573	−4.8669	0.0069703	SNRNP40
circ_0006240	4.5803	0.012792	BBS9	circ_0002342	−4.8618	0.0069977	LSG1
circ_0009110	4.5659	0.013195	FAM169A	circ_0015293	−4.835	0.011635	C4BPA
circ_0008285	4.5506	0.013832	SRFBP1	circ_0015483	−4.8146	0.0077605	NKAP
circ_0015586	4.4912	0.019519	PIR	circ_0000778	−4.8001	0.0079754	ERCC6L2
circ_0013267	4.4627	0.016911	SLA-DQB1	circ_0003666	−4.7498	0.0088787	PDCD4
circ_0013254	4.4324	0.021108	SLA-1	circ_0007960	−4.7383	0.009741	QKI
circ_0000110	4.4283	0.017194	ASAP3	circ_0002828	−4.7326	0.0092144	ITGA9
circ_0000726	4.4093	0.018716	DENND1B	circ_0005090	−4.7231	0.0094609	BAZ2B
circ_0001632	4.4093	0.018716	MPP2	circ_0000257	−4.714	0.0098126	ATP8B1
circ_0015288	4.4059	0.018032	SRGAP2	circ_0010838	−4.6454	0.011096	RAD54B
circ_0010525	4.3778	0.02352	BCAR3	circ_0002634	−4.6439	0.011083	LTN1
circ_0011115	4.3745	0.01908	FBXO7	circ_0001669	−4.6043	0.012109	TOP2A

### Validation of circRNAs using RT-PCR

**Fig. 2:**
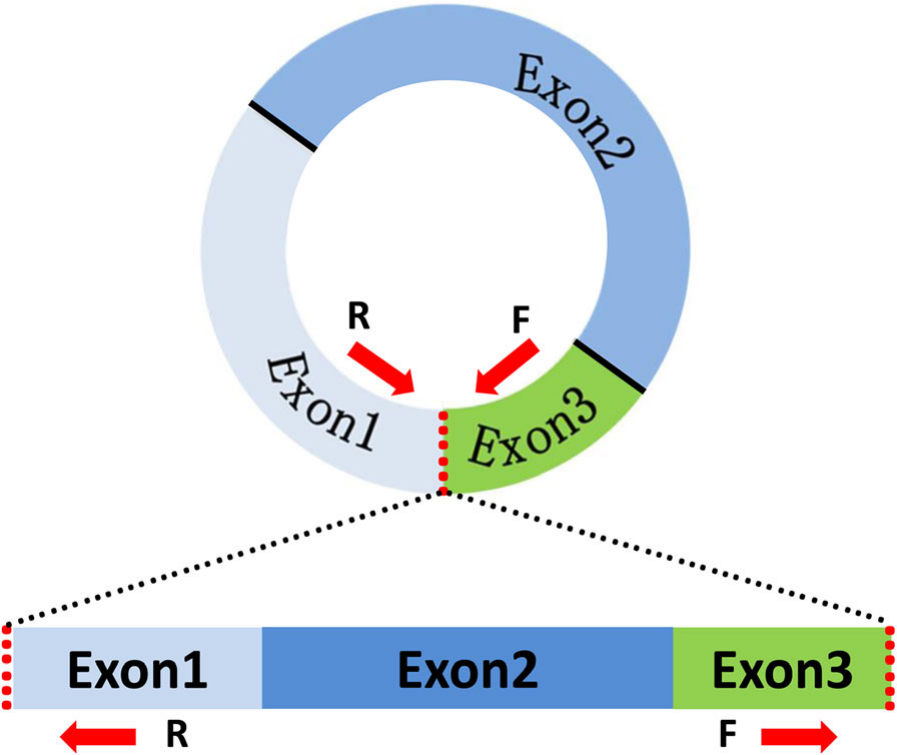
Circular junctions were amplified using divergent primers. Red arrows indicate the divergent primers

**Fig. 3:**
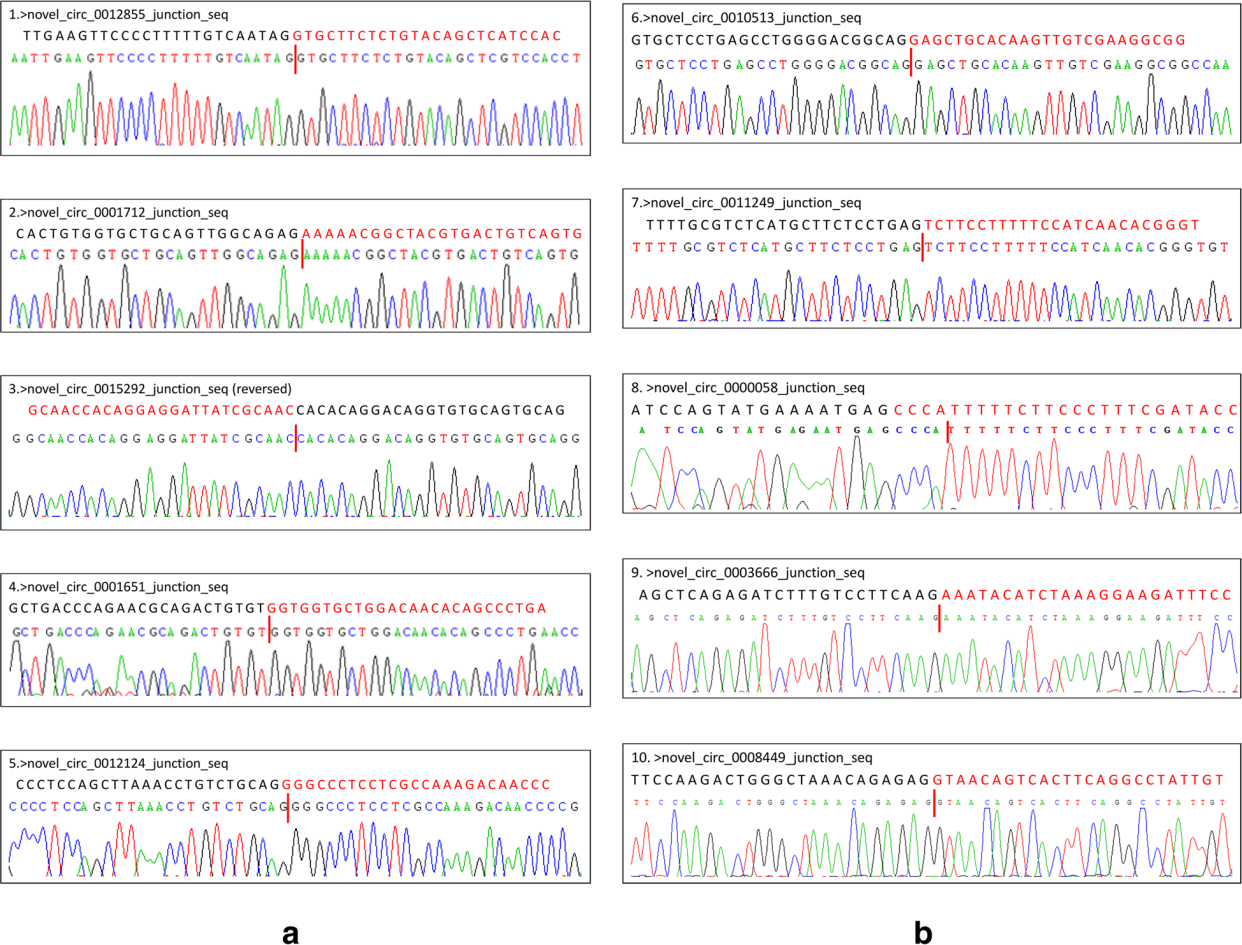
DNA sequencing of each RT-PCR product amplified using divergent primers identifies circRNA back splicing sites. **a** Circ_0012855, circ_0001712, circ_0015292, circ_0001651 and circ_0012124; **b** circ_0010513, circ_0011249, circ_0000058, circ_0003666, and circ_0008449

**Fig. 4:**
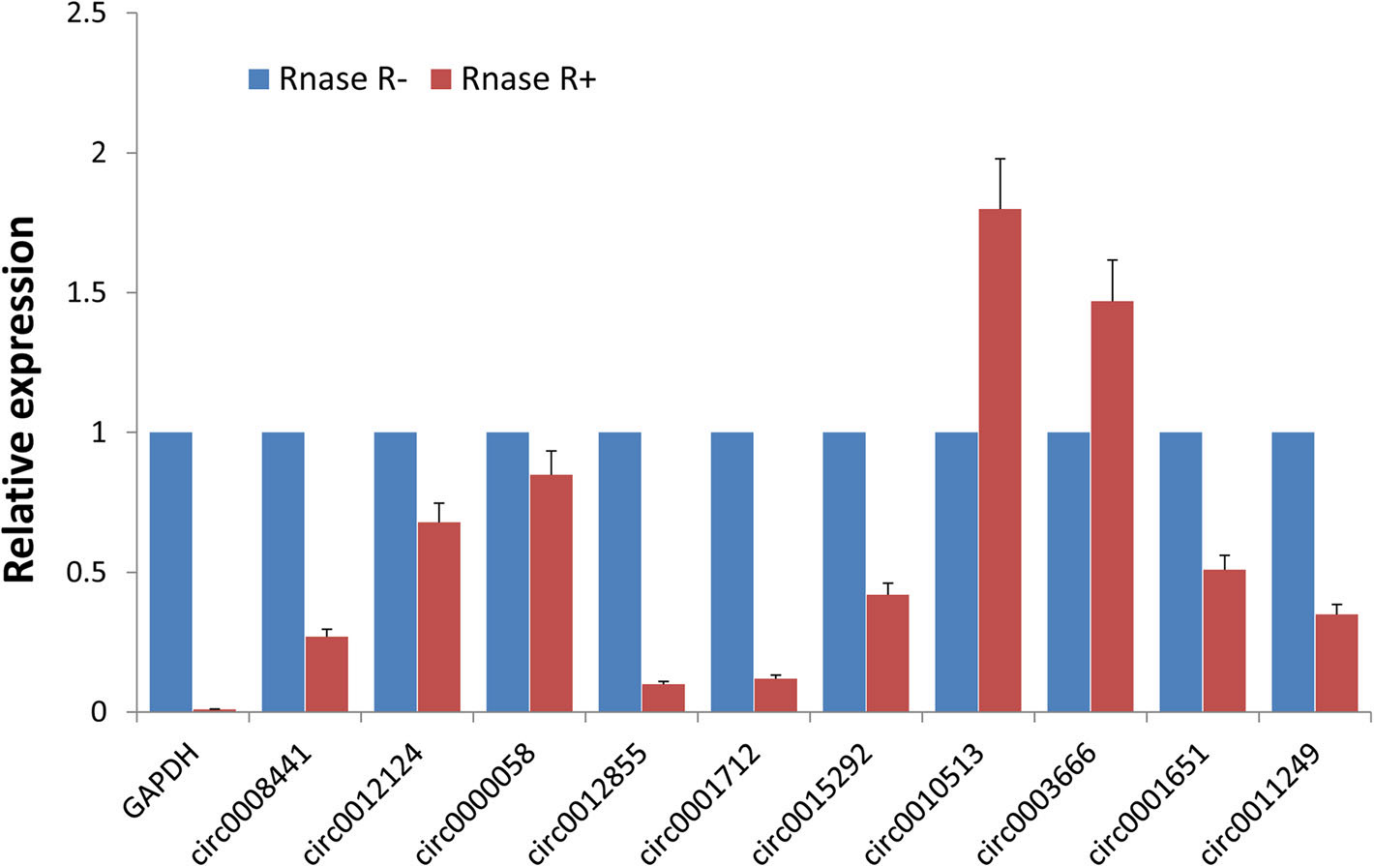
Determination of resistance to RNase R digestion using qPCR. GAPDH was used as a linear control

To further validate the RNA-seq findings, RT-PCR was employed and specific primers were designed to reverse amplify the circRNA junctions (Fig. 2). For the 10 randomly selected circRNAs, the head-to-tail junction sites were quantified via RT-PCR analysis and confirmed using DNA sequencing (Fig. 3). Additionally, the resistance of the circRNAs to RNase R digestion was also examined using qPCR. All of the examined circRNAs were found to be RNase R resistant, while the internal control, GAPDH, was not detected (sensitive to RNase R; Fig. 4).

### Verification and analysis of differentially expressed circRNA

**Fig. 5:**
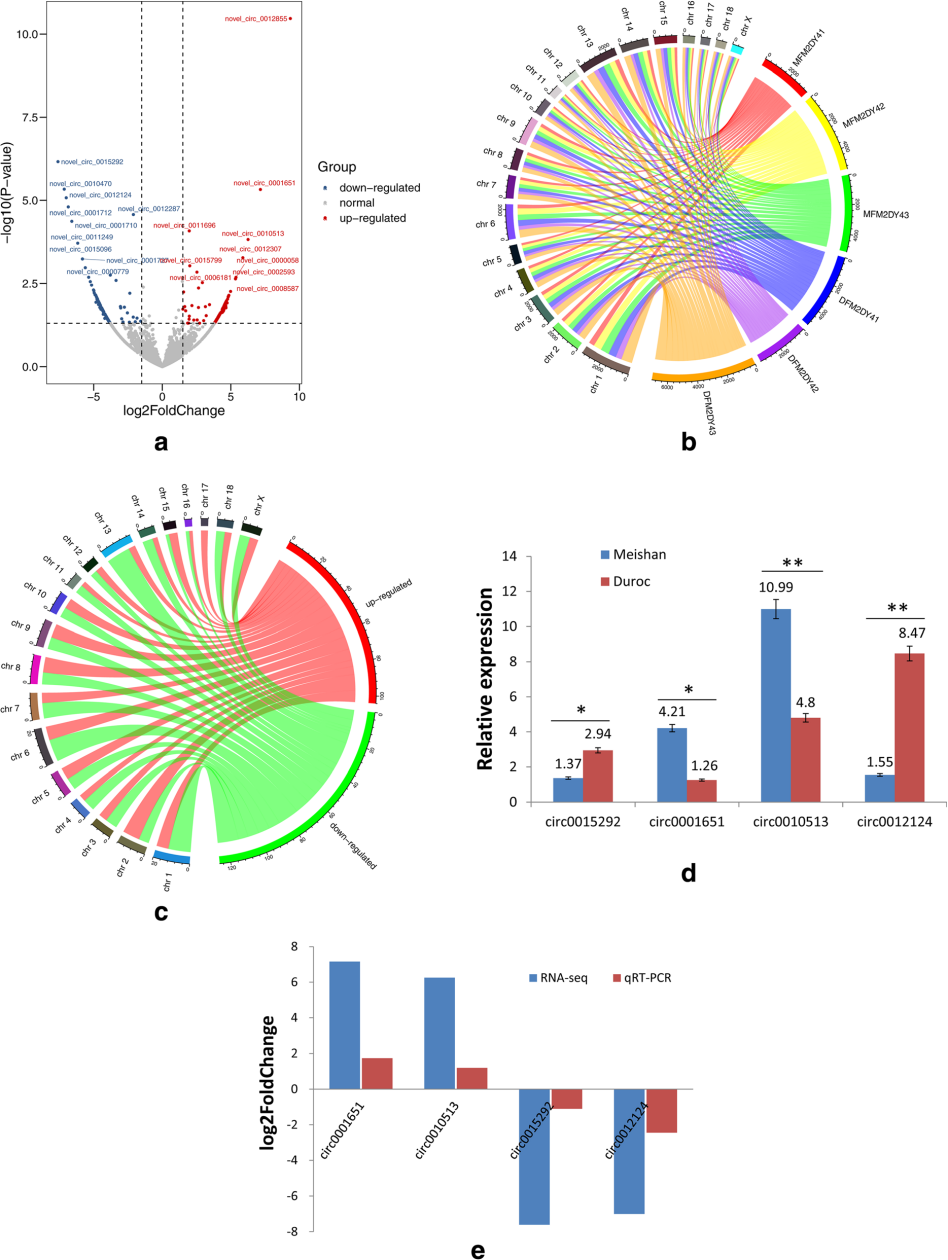
Analysis and validation of differentially expressed circRNAs in Meishan ovarian follicle samples relative to Duroc samples. **a** Volcano plot demonstrating a distinguishable circRNA expression pattern between Meishan and Duroc ovarian follicles; **b** Circos plot displaying circRNA chromosomal distributions; **c** Circos plot displaying differentially expressed circRNA chromosomal distributions; **d** Relative expression levels of a subset of four circRNAs; **e** Comparison of qPCR and RNA-seq results confirms a high degree of consistency

Further analysis confirmed the 244 differentially expressed middle follicle circRNAs, 111 up-regulated and 133 down-regulated, in Meishan sows relative to Duroc sows (Fig. 5a). Many of the circRNAs were distributed across various chromosomes, with many located on chromosome 1 (Fig. 5b). Next, four differentially expressed circRNAs were selected, and their expression levels were quantified via qPCR. The results showed in Meishan follicle samples that circ**_**0001651 and circ**_**0010513 expression is up-regulated relative to Duroc samples, while circ**_**0015292 and circ**_**0012124 expression is down-regulated (Fig. 5d). These findings are consistent with the RNA-seq findings and confirm that the sequencing results are accurate and reliable (Fig. 5e).

### GO and KEGG enrichment analysis of differentially expressed circRNA host genes

**Fig. 6:**
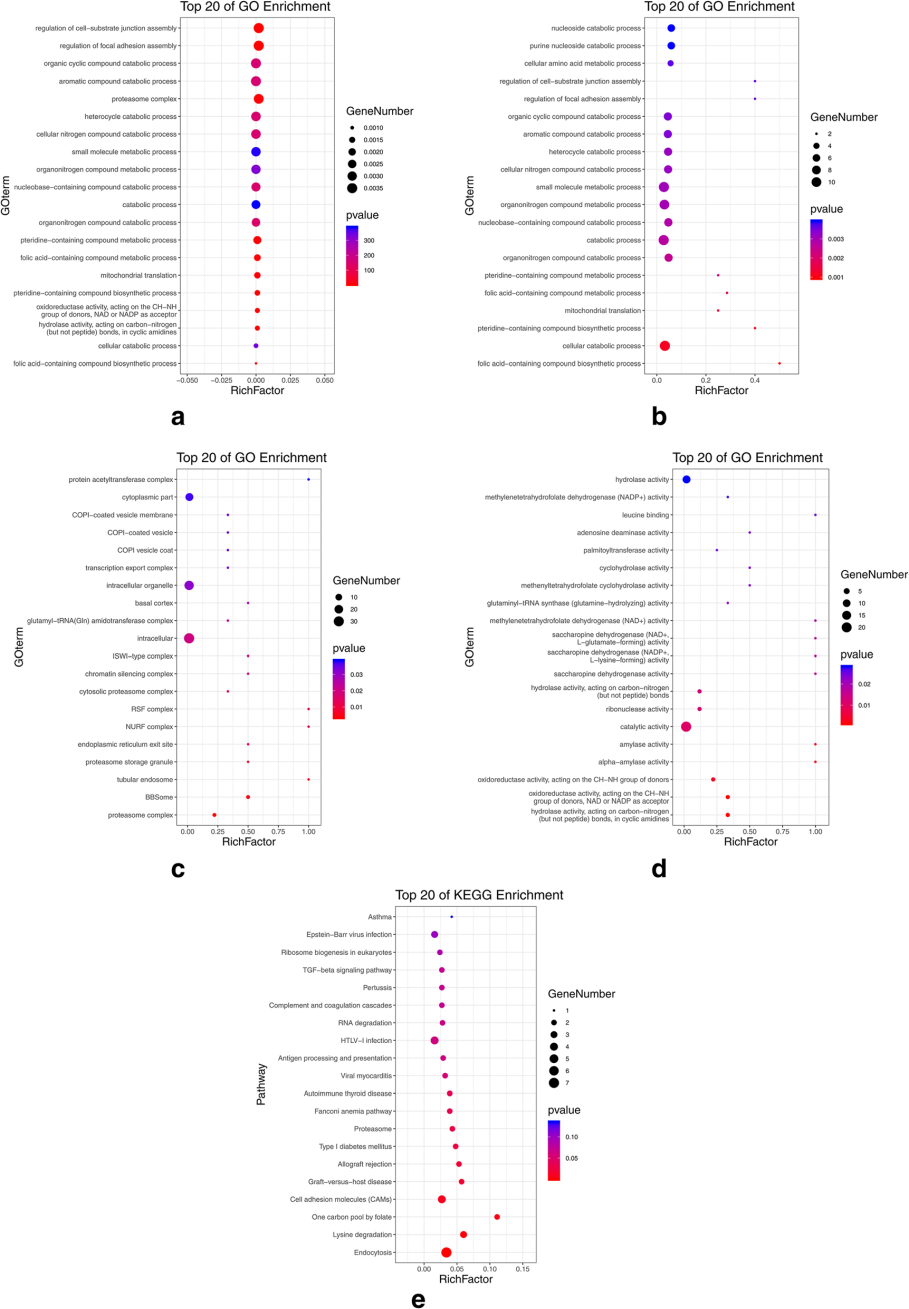
Enrichment analysis of host genes associated with the differentially expressed circRNAs. **a** Top 20 GO terms overall; **b** Top 20 GO terms in biological processes; **c** Top 20 GO terms in cellular components; **d** Top 20 GO terms in molecular functions; and **e** Top 20 KEGG pathways

In previous studies, circRNAs have been shown to regulate the expression of their host genes [34–36], and their functions may relate to those of their host genes. Therefore, GO and KEGG pathway enrichment analysis was performed using the host genes associated with the differentially expressed circRNA. GO analysis identified 201 significantly enriched terms within the biological processes, molecular functions, and cellular components groups. The top 20 GO terms were associated with several functional categories, including metabolic processes, intracellular protease complexes, and enzymatic activity (Fig. 6), thus indicating that some circRNAs are involved in the basic biological regulation of porcine follicular development. The KEGG pathway analysis enriched 87 pathways (Fig. 6e), including PI3K-Akt, oocyte meiosis, and TGFβ-SMAD signaling pathways that are involved in follicular granulosa cell growth regulation. These results indicate that circRNAs play an important role in the formation and development of porcine follicles.

### Porcine follicle circRNA functional predictions

**Fig. 7:**
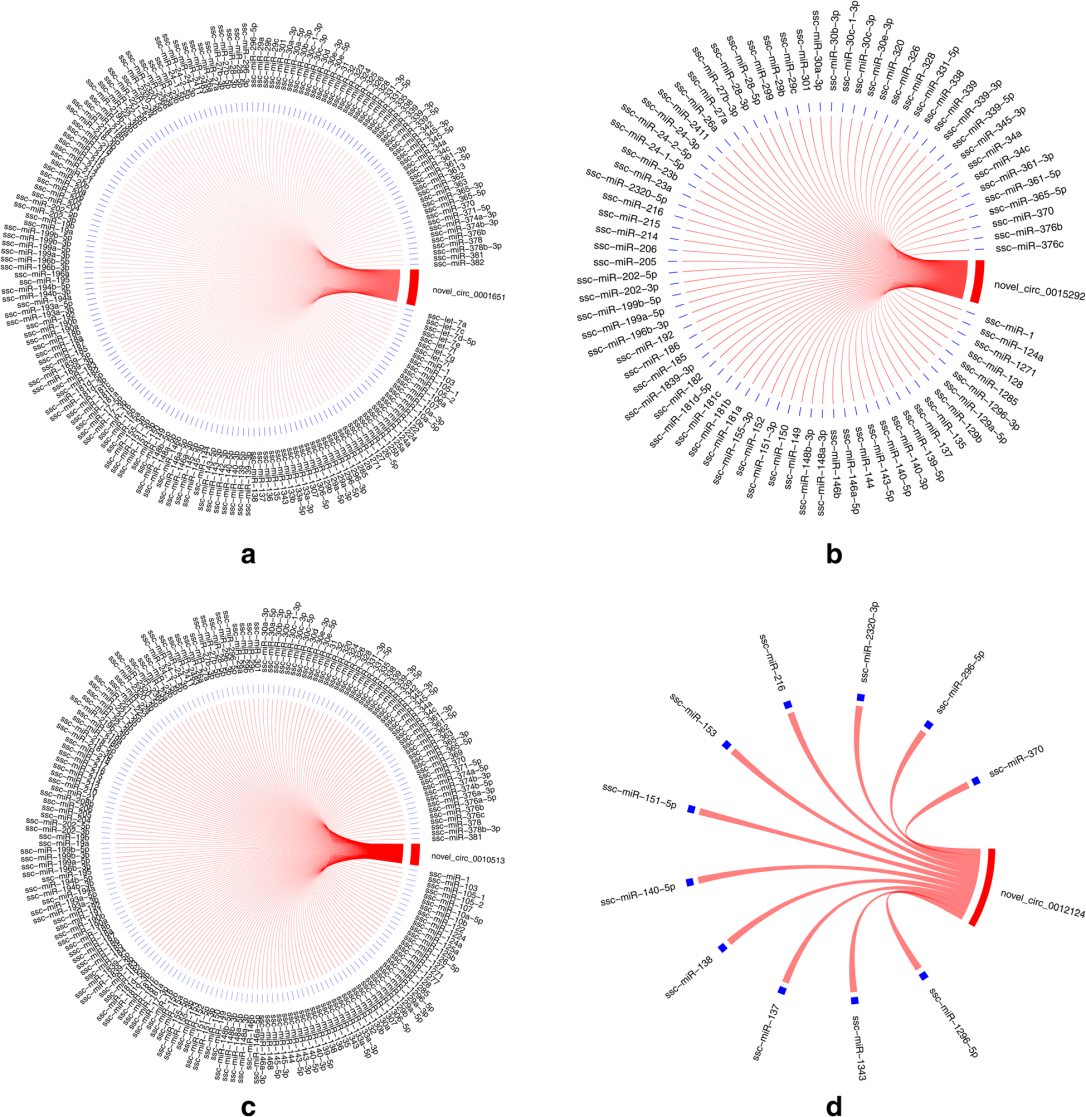
CircRNA–miRNA correlation networks for four circRNAs and their target miRNAs. **a** Circ_0001651-miRNA; **b** circ_0015292-miRNA; **c** circ_0010513-miRNA; and **d** circ_0012124-miRNA

**Fig. 8:**
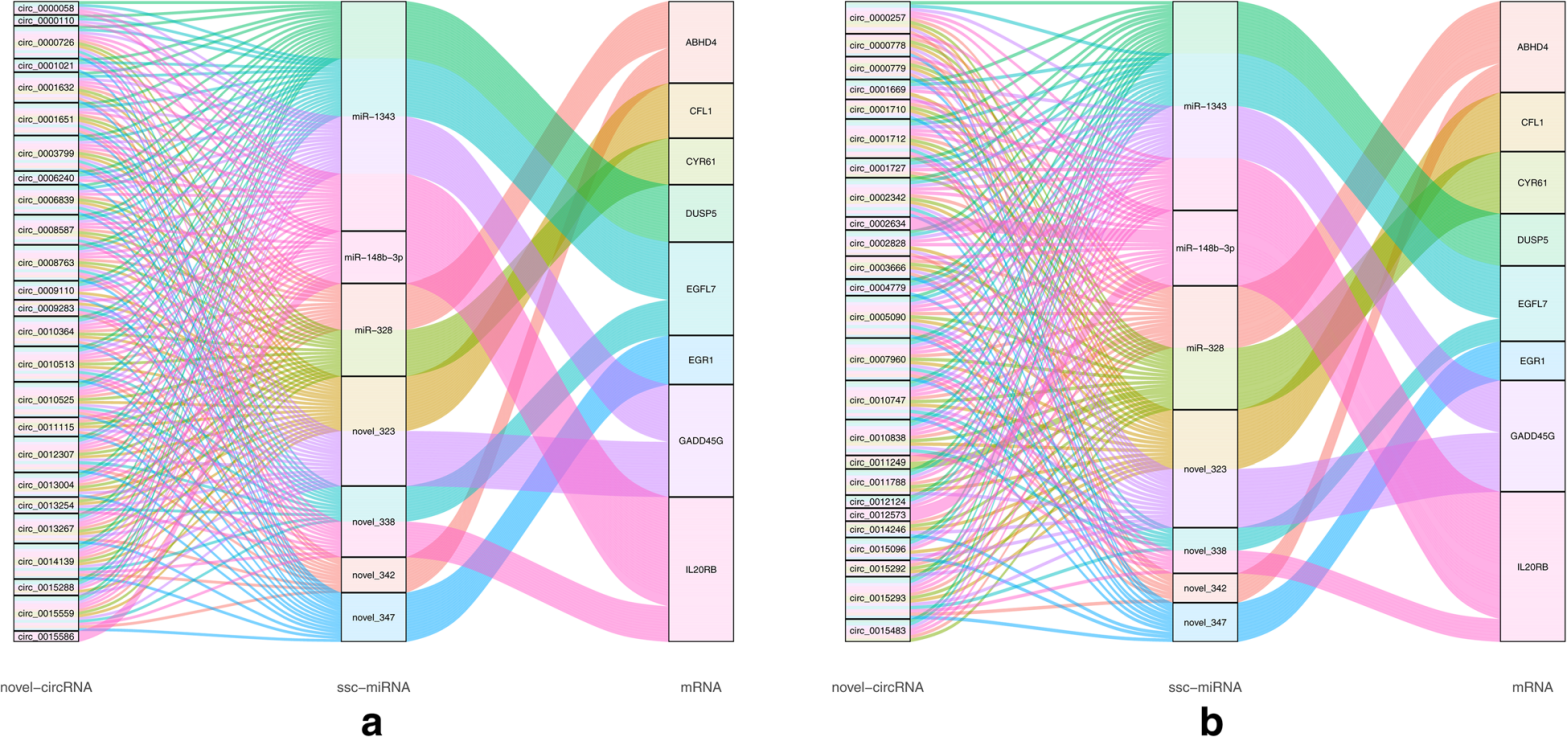
Sankey ceRNA network diagram for the top 25 differentially expressed ovarian follicle circRNAs. Each rectangle represents a gene, and the connection degree of each gene is visualized based on the size of the rectangle. **a** The top 25 up-regulated; **b** the top 25 down-regulated

Previous studies have suggested that circRNAs can act as a miRNA sponge, thereby affecting the expression of miRNA target genes [2, 24, 37, 38]. Herein, miRanda and psRobot software were used to analyze potential interactions between circRNAs and miRNAs, with 1,925,007 potential interactions identified between 15,866 circRNAs and various miRNAs. Moreover, it is worth noting that some of the known miRNAs are closely related to follicular development and are considered prospects for future research. The circRNAs examined in this study were found to contain multiple conserved binding sites for miRNAs, such as miR-21, miR-144, or miR-181-a, which indicated that circRNAs are involved in follicular development (Fig. 7). To elucidate the functional roles of the examined circRNAs in association with miRNAs, circRNA target miRNAs and downstream regulated mRNAs were predicted, and a basic circRNA-miRNA-mRNA connective network was established (Fig. 8). The results indicated that porcine follicular growth and development are likely to be affected by circRNAs.

## Discussion and conclusions

CircRNAs are a new class of endogenous non-coding RNAs that were once considered a by-product of splicing errors but have been found to be widely expressed in human cells and function in many biological processes [7, 39]. Furthermore, studies have shown that circRNAs are involved in regulation [40–42] and can be associated with diseases, including cancer [43, 44]. CircRNAs are highly conserved and very stable, contain tissue-specific sequences, and contain unique ceRNA features [2, 24, 45]. Moreover, studies have suggested that human and mouse early embryos have a high degree of similarity in relevant biological processes where circRNA host genes appear to be primary factors [19, 46]. Among different species, most circRNA expressions are highly conserved [45, 47], with abnormal circRNA expression associated with many human diseases, such as cancer, nervous system diseases, and cardiovascular diseases [21, 43, 48, 49]. In ovarian cancer cells, the up-regulation of hsa-circ-0061140 promotes EMT, cell proliferation, and migration [50]. However, when it comes to livestock, especially swine, reproduction-associated circRNA expression remains unclear. Thus, this study focused on exploring the potential role of circRNAs in porcine follicle development. First, RNA-seq was utilized to establish follicular circRNA profiles for Meishan and Duroc sows. A total of 15,866 circRNAs were identified, with 244 being differentially expressed (111 up-regulated and 133 down-regulated).

At present, research focused on examining the regulation of circRNAs in animal reproduction has been making small gains year by year. To improve the reproductive capacity in sows, it is important to more fully characterize the follicles and the factors that influence them. During oocyte maturation and early embryo development, granulocytes (GCs) are very important, and follicular atresia results in granulosa cell apoptosis [51, 52]. Dicer is a conserved ribonuclease and plays a key role in regulating oocyte development in mice [53]. Furthermore, mir-145 can inhibit the proliferation of mouse granulosa cells by targeting a gene called activin receptor IB (*ACVR1B*) [54]. In a previous study examining human GCs, circRNA_104816 and circRNA_103827 were found to potentially serve as biomarkers indicating follicular microenvironment damage, with their up-regulation being closely associated with a decreased ovarian reserve and poor reproductive outcomes [55]. Furthermore, in another study examining goat pre-ovulatory follicles, 37 differentially expressed circRNAs were identified, with chi-circ 0008219 found to regulate follicular growth by modulating three miRNAs [28]. Based on the above results, we hypothesized that circRNAs may serve as novel regulators of ovarian follicle growth and development during porcine reproduction.

Some studies have shown that circRNAs can modulate miRNAs by acting as a miRNA sponge [7, 24, 56]. In cancers, CDR1as has been shown to act as a sponge for mir-7 and subsequently suppresses its activity and promotes tumor development [57]. Moreover, testis-specific sex-determining region Y (SRY) 9 acts as a sponge for mir-138 and contains 16 mir-138 binding sites, thus reducing its effects [24, 58]. Herein, miRanda and PSrobot were used to predict miRNA target sites within the identified porcine follicular circRNAs. Of the identified miRNA interactions, miR-191, miR-210, miR-132, miR-370, and miR-181-a were found to be associated with follicular development. Furthermore, the results showed that a single circRNA has different target binding sites for different miRNAs. One of the circRNAs, ssc-circ-0001651, was found to contain 18 potential binding sites for 8 different miRNAs associated with follicular granule cell development (partial results only), including let-7 g (3 target sites), miR-21 (1 target site), miR − 224 (1 target site), miR-10b (4 target sites), miR-16 (4 target sites), miR-106a (1 target site), miR-19b (2 targets sites), and miR-31 (2 target sites). For ssc-circ-0015292, 8 target sites were identified (partial results only) that bind miR-34a (1 target site), miR-144 (1 target site), miR-320 (1 target site), miR-181a (1 target site), miR-150 (1 target site), miR-23a (2 target sites), and miR-27a (1 target site). Therefore, these findings suggest that ssc_circ_0001651 and ssc_circ_0015292 can act as potential ceRNAs, which would make them newly identified porcine ovarian follicular development regulators, but further investigation is required.

In addition to regulating gene expression, circRNAs have also been shown to serve other functions. Recent studies have indicated that circRNAs can direct protein synthesis via mRNA modulation, and a few of them may be converted to proteins via an IRES (internal ribosome entry site) insertion [59, 60]. Several factors and pathways are known to be involved in follicular growth and development, including follicle-stimulating hormone (FSH), insulin growth factor (IGF), and transforming growth factor-β (TGFβ) and their related receptor-mediated signaling pathways, including PI3K-Akt, Wnt/β-catenin, and TGFβ-SMAD signaling pathways. Furthermore, Tao et al. reported that prior to goat ovulation, the host genes of ovarian follicle circRNAs participate in ovarian corpus callosum generation pathways and p53 signaling [28]. CircRNA host genes have also been implicated in the production of ovarian steroids and their mediated signals that are critical for biological processes such as follicular growth, oocyte maturation, and ovulation [61].

## Conclusions

In this study, GO and KEGG pathway annotations identified important biological processes and pathways, including metabolic processes, enzyme activity regulation, endocytosis, steroid hormone biosynthesis, cell cycle and cell adhesion, and homologous recombination. Moreover, important signaling pathways, such as TGF-β, p53, insulin, oocyte meiosis, and PI3K-Akt signaling pathways, were enriched. Collectively, these findings suggest that circRNAs can affect the development of porcine follicles by modulating associated pathways.

In summary, ovarian follicle circRNA profiles were obtained for Meishan and Duroc sows, with differentially expressed circRNAs also identified. GO and KEGG analyses were then utilized to elucidate the roles of the identified differential circRNAs, with several found to be involved in ovarian follicle growth and development regulation. This study provides further insight into the mechanisms of porcine follicle development and the roles of circRNAs.

## Data Availability

The datasets and supporting conclusions are included within this manuscript or its supporting files. The datasets generated during this study are available from the corresponding author upon request.
